# Supplementary material for: The interactome of intact mitochondria by cross-linking mass spectrometry provides evidence for coexisting respiratory supercomplexes
Source: Mol Cell Proteomics. 2017 Dec 8;17(2):216–32. doi: 10.1074/mcp.RA117.000470 (PMC5795388; doi:10.1074/mcp.RA117.000470)
Supplement: Supplemental Data [file supp_RA117.000470_133922_0_supp_23978_fzffwf.zip › spectra_annotation/mito_DR_spectra_annotation/371-1-4-1-17-1.pdf]

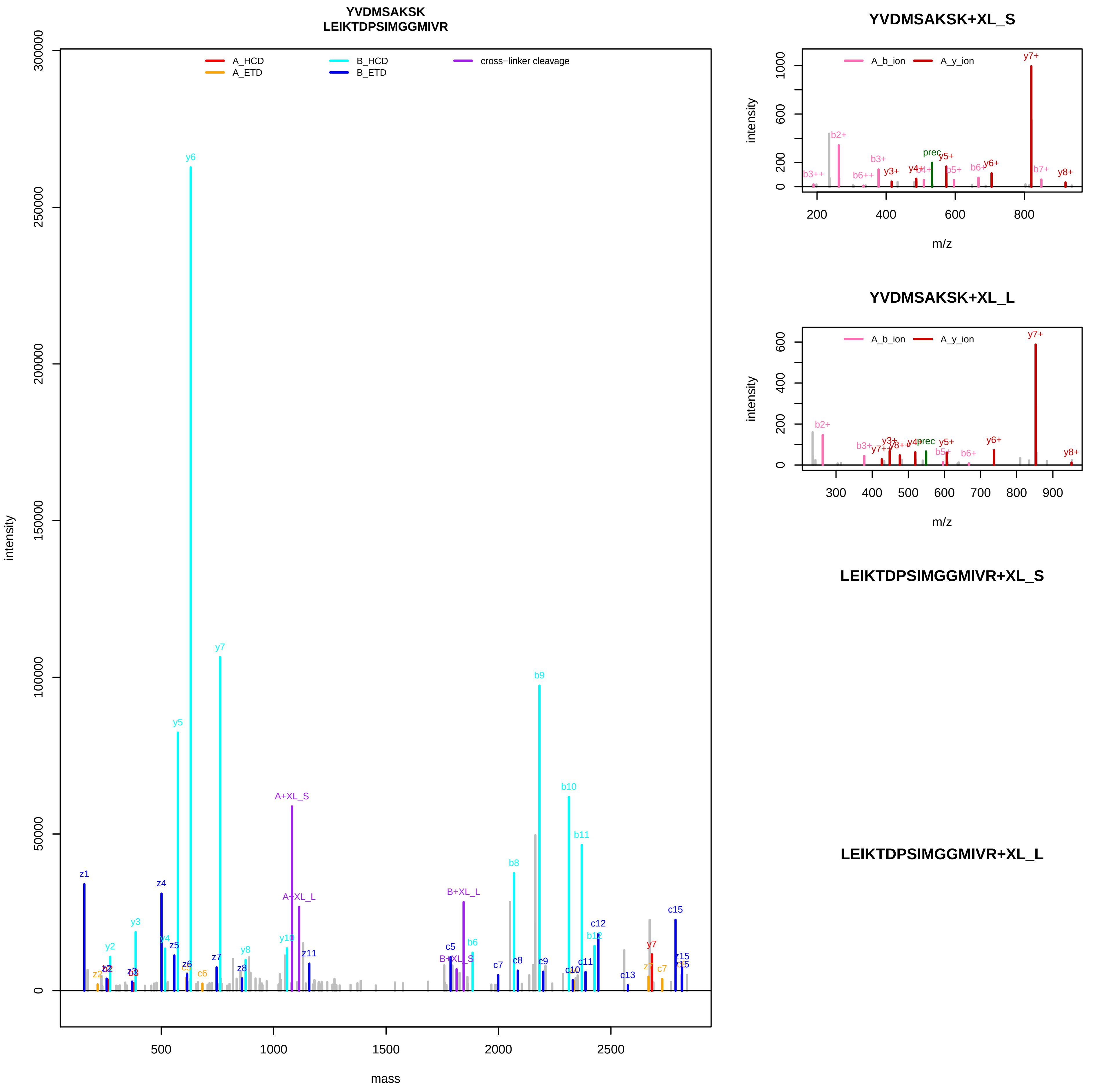

YVDMSAKSK
LEIKTDPSIMGGMIVR
A_HCD
A_ETD
B_HCD
B_ETD
cross−linker cleavage
intensity
mass
0
50000
100000
150000
200000
250000
300000
500
1000
1500
2000
2500
y6
y7
b9
y5
b10
b11
A+XL_S
A+XL_L
B+XL_L
B+XL_S
b8
z1
z4
c15
c12
YVDMSAKSK+XL_S
YVDMSAKSK+XL_L
A_b_ion
A_y_ion
m/z
prec
y7+
LEIKTDPSIMGGMIVR+XL_S
LEIKTDPSIMGGMIVR+XL_L
